# Perceptions about mercury and lead in fish consumed in Lake Albert fishing communities Uganda

**DOI:** 10.1080/23311932.2016.1220344

**Published:** 2016-08-17

**Authors:** Tamale Andrew, Ejobi Francis, Muyanja Charles, Irene Naigaga, Nakavuma Jesca, Ocaido Micheal, Katuhoire Anne, Amulen Deborah

**Affiliations:** ^a^ College of Veterinary Medicine, Animal Resources and Biosecurity, Makerere University, P.O. Box. 7062, Kampala, Uganda; ^b^ Department of Food Technology and Nutrition, MakerereUniversity, P.O Box 7062, Kampala, Uganda; ^c^ College of Health Sciences, Makerere University, P.O. Box. 7062, Kampala, Uganda; ^d^ Department of Crop protection, Ghent University, Ghent, Belgium; ^e^ Middle East Technical University, Turkey

**Keywords:** individual perceptions, vulnerable community, fish consumption, heavy metals, benefits, risks, contamination

## Abstract

Fish consumption is a lifestyle in fishing communities influenced by individual and communal perceptions. However, information about individual perceptions about fish consumption in the vulnerable fishing community in a developing country is lacking. Without this study, the benefits of fish consumption in a vulnerable community may not be realized. Data collection was executed using key informant interviews and survey structured questionnaires. The key informants include fisheries, community development, veterinary, community and environmental officers. The household heads were the respondents. The Qualitative data was organized and queried using QSR Nvivo 10 and quantitative data analyzed with SPSS version 22. The perceived benefits of eating fish are health, income, nutrition and manhood. The perceived risks are Stigma and ill health. The factors increasing fish consumption are heedless of fish consumption benefits (*p* = 0.041) and household size i.e. number of adults more than seven (*p* = 0.020). Those decreasing are methods of preparation of fish i.e. boiling and frying (*p* = 0.019 and *p* = 0.010) and oblivious about organizations dealing with fishing activities (*p* = 0.029). An awareness campaign is needed to demystify the health benefits and fallacies of fish consumption. The knowledge on individual perceptions associated with fish consumption will increase fish consumption but with fewer risks.

## 1. Introduction

Fish consumption has negative and positive consequences in the indigenous population and more specifically in pregnant women, children and adults as documented by Power, Klein, Guiguer, and Kwan (2002). The benefits of fish consumption include reduction of cardiovascular diseases, boosting body immunity and provision of proteins (Olmedo et al., 2013). Increased fish consumption is also unfortunately associated with uptake of contaminated fish (Teisl, Fromberg, Smith, Boyle, & Engelberth, 2011). The major risk associated with fish consumption is that of heavy metals uptake especially mercury and lead (Petre, Sackett, & Aday, 2012). To counteract the heavy metal consequences, governments worldwide institute fish consumption advisories (Burger & Gochfeld, 2008; Engelberth et al., 2013; Teisl et al., 2011). The fish consumption advisories create awareness about the risks and benefits associated with fish consumption (Burger & Gochfeld, 2008). The message disseminated to communities focuses on the direct and indirect effects of the guidelines (Burger, 2000; Carvalho, Matos, Mateus, Santos, & Batoréu, 2008; Mariën & Patrick, 2001; Oken et al., 2012; Wheatley & Wheatley, 2000). The indirect effects include community welfare, attitudes, behavior change, individual factors, and location. While the direct effects target the consequences, choice of interventions and target group.

Regardless of the community: Vulnerable or non-vulnerable, the perceptions linked to fish consumption are either individual or communal or both. The individual perceptions are classified into perceived benefits or risks. These individual perceptions either directly or indirectly affect fish consumption. The three construct model exhibited by Pieniak, Verbeke, Scholderer, Brunsø, and Ottar Olsen (2008) in five European countries showed that people who eat healthy foods have a high affinity for fish consumption, those who know about risks of fish consumption decreased feeding. Persons in good health had no correlation with fish consumption. The study pointed out that sociocultural and individual aspects that affect fish consumption should be considered if the benefits of fish consumption are to be realized.

The perceptions which increased fish consumption include age, nutrition and source of information. Different groups in the USA like pregnant women, women of childbearing age, children less than 16 years of age, elderly and adolescents have different perceptions regarding fish consumption. The perception of the elderly was that they obtained more cardiovascular benefits from boiled fish than fried fish. However, the children are a crucial age group for the fishing community since the hazards affect them fivefold as compared to adults (Kuntz, Ricco, Hill, & Anderko, 2010). Regarding nutrition, adolescents from Cameroon depend on the nutrition benefits from fish to appear beautiful and increase the body size of the woman (Bloomingdale et al., 2010; Cohen et al., 2005; Dapi, Omoloko, Janlert, Dahlgren, & Håglin, 2007; Mozaffarian et al., 2003)

The perceptions which lead to risks amongst fishing community are vulnerability, message, information available, frameworks and theories. Vulnerable groups in Africa have nutritional and livelihood challenges related to the sales of the fish to developed countries or income diversification to get the much-needed income or even still have transactional sex for fish stock or exchanged sex for fish (Béné & Merten, 2008). Furthermore, consumers who are not vulnerable have perceptions about fish consumption too i.e. undeclared risks of fish consumption, the role of social media, low adoption to new products and individual factors which affect consumption (Béné & Merten, 2008; Geheb et al., 2008; Pieniak, Verbeke, & Scholderer, 2010).

Abstract messages stopped women of childbearing age from fish consumption while in the Russian study the message was linked to safety of the products consumed (Barnett et al., 2011; Grunert, 2005; Ruxton, 2011; Ueland et al., 2012; van Dijk, Fischer, Honkanen, & Frewer, 2011; van Kleef et al., 2006; Verbeke, Vanhonacker, Sioen, Van Camp, & De Henauw, 2007)

Parents of children and newborns in the USA need information for fish risk to come from health care providers if it is to be trusted (Kuntz et al., 2010). While the students in the Netherlands showed that benefit and risk are related to information availed, the level of the commonness of the fish and psychology of the consumer. This information probably explains the difficulty in behavior change without the right information and frame of mind.

The frameworks, models, and theories which have attempted to explain the perceived risks and benefits associated with consumption of foods including fish included two-pronged and attitude frameworks and grounded, miserabilism, economic and value behavior theories (Costa-Font & Mossialos, 2007; Pieniak et al., 2008; Teisl, Fein, & Levy, 2009; Wohl, 1998). The theoretical perspectives linked to fish consumption perceptions in Africa, Europe, and the USA spoke openly of poverty amongst the adolescents in Cameroon and the fishing community in East Africa while talking about the lack of purchasing power and behavior changes in Spanish and American consumers (Béné & Merten, 2008; Dapi et al., 2007; Teisl et al., 2009; Tudoran, Olsen, & Dopico, 2009). The theoretical perspectives guiding the current study are the Vygotsky theory which looks at behavior change in the community in light of the information provided, language used and societal discernment of the meaning and the cultural consensus theory which looks at the systematic distribution of information and knowledge in the community (Anders & Batchelder, 2013; Yasnitsky & Ferrari, 2008).

Research on individual perceptions about fish consumption is extensively covered between the late nineties and the early twenty first century in Europe and North America with only limited contribution from Asia and Africa. In the East African region, there is only one study by Geheb et al. (2008) who concentrated on the linkage between fish exports and malnutrition in the fishing communities. The previous study, however, did not look at the fishing community perceptions on fish consumption, a gap the current study is going to address.

The study was carried out in the Albertine region Uganda. This area is characterized by man-made sources of lead and mercury include: Oil exploration, poor oil waste management arising from leaving behind pools of unutilized crude oil which are washed down stream into the lake and charcoal burning (Hindrum, 2011). Attempts are being made by the Ugandan government to prevent disastrous effects attributed to lead and mercury in the Lake Albert region. The measures instituted to lower the man-made sources of the two metals in the lake include Environment impact assessment, alternative sources of energy and sensitization (Campbell, Dixon, & Hecky, 2003). Nonetheless, these attempts can only achieve the desired effects in the long term. The available short term measure is to assess and report the hazards present in the common fish consumed and the associated individual perceptions (Petre et al., 2012).

It is vital that the study of individual perceptions about fish consumption in fishing communities be carried out if the benefits of fish consumption are to be harnessed.

## 2. Methods

This perception study stance form is the post positivist paradigm, uses mixed methods and the convergent parallel approach (Creswell, 2013). The methods employed are key informant interviews and surveys (Creswell, 2013). Data collection occurred from March to June 2015.

### 2.1. Study area

This perception study was carried out in Lake Albert fishing communities in Uganda. Lake Albert is located in western Uganda and is located 1.52 N; 30.86 E 10 km and is the lake that occupies the most north part of the rift valley (Karp, Scholz, & McGlue, 2012). The study was carried out in Hoima district in the four sub counties of Kyangware, Kabwoya, Buseruka and Kigolobya and these are the only sub counties with access to Lake Albert waters. The corresponding landing sites of Buhuka, Nkondo, Kaiso Tonya and Kibiro respectively. The area is characterized by oil exploration, charcoal burning, fishing, a world center of biodiversity and wildlife heritage site. The highly populated area has some influx immigrants for livelihood. Hoima residents live in abject poverty with less than 2% access to electricity, and the majority depend on subsistence agriculture (Hindrum, 2011). The fishing communities are multicultural i.e. have natives from Uganda and Democratic Republic of Congo. This cultural diversity alters or modifies the inherent fishing practices and lifestyles around the fishing community.

### 2.2. Participant selection

The key informants were purposively selected based on their level of knowledge about the fish consumption and environment and their leadership roles in their respective departments in the study district. These informants apart from the local leadership are all under the production department and this is mandated by law to handle all the district issues on environment related research, community interventions, agricultural production and planning. So these were the persons who will implement the findings of the research.

Households chosen for the questionnaires administration were those found around Lake Albert fishing community less than or equal to 2 km from the landing site.

### 2.3. Sample size for the survey

The sample size for the cross-sectional household survey was determined by the formula by sample power 3 IBM SPSS software for a population.

$$n=z\alpha_{2}\;\text{PQ}/d_{2}$$

Where *n* is the sample size of households, *Zα* is the *z* value at *α* = 0.05 level of significance, *P* is the expected prevalence of the condition in the population under study, *Q* is 1−*P, d* is the desired error of the estimate (Thrushfield, 1995).

A total of 384 Households is considered sufficient for the study. However, this number was not realized since the households on the four landing sites on Lake Albert were about 500 and this was less than that anticipated therefore a finite factor for the correction

New sample size = sample size/1 + (sample size−1/Population) (Thrushfield, 1995)

This gave a new sample size of 217 Households. Due to non-responses, an anticipated 20% was factored for the sample size bringing the total households to 260. However, the study collected data from 273 households. These were selected randomly from households around the landing sites.

### 2.4. Data collection

#### 2.4.1. Qualitative

The five key informant interviews elicited data from fisheries, community development, community, environmental and veterinary officers. The tool used for this key informant interview was an unstructured interview guide (Moretti et al., 2011). This provided data on the perceived benefits and risks associated with fish consumption. The key informant interviews took between 45 min to 1 hour and individual consent was sought from each officer before the study commenced. The data was recorded in addition to the notes taken during the interview.

#### 2.4.2. Quantitative

The household survey used a structured questionnaire for data collection from households. The interviewers were trained in administration of the tool (structured questionnaire). The questionnaires were pretested and modified for reliability and then finally administered. A total of 273 questionnaires were administered for the study.

### 2.5. Data analysis

The data collection and analysis was separate for each component. Triangulation of the findings occurred at the discussion stage. More weight was placed on the qualitative than quantitative aspect.

The qualitative data was transcribed and then word processed. The word processed transcripts were uploaded into QSR Nvivo 10 software and then coded deductively. Thereafter, relationships were established, themes generated and visualized.

The quantitative data was exported to SPSS version 20 for Descriptive analysis. This generated summary statistics, tables of results and graphs. Then Chi square analysis was conducted to establish the perceptions associated with fish consumption. The interpretations were made using level of significance *α* = 0.05 and Confidence intervals of 95%.

A general linearized model for the Poisson family was run to establish contribution of the factors to fish consumption. This then was reported using narrative, figures /graphs /tables and levels of association between variables.

### 2.6. Ethical considerations

This research was reviewed and approved by institutional ethical review board of College of Veterinary Medicine, Animal Resources and Biosecurity Makerere University under record number V:AB:REC/15/103. The research was also approved by the Ugandan National Council of Science and Technology under SIR 140

## 3. Results and discussion

### 3.1. Quantitative

#### 3.1.1. Demographic characteristics of the respondents

**Figure 1.**
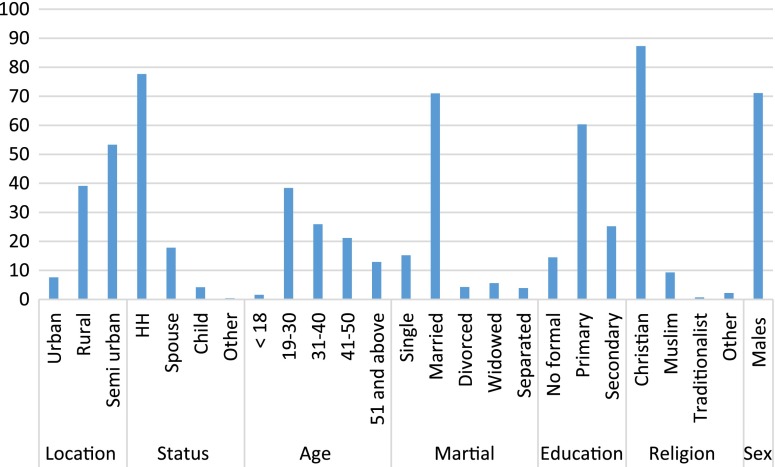
Demographic attributes of respondents in Lake Albert.

The population at Lake Albert was relatively young since of the households studied, the majority were aged between 19–30 years. However, 70% of the respondents were married and the majority had children. Most had minimal education i.e. Primary level of education and none had tertiary education. The majority of the household heads were males a feature typical of paternalistic families. The distribution of the demographics is displayed in Figure 1.

#### 3.1.2. Fish consumption attributes of the respondents

Like in all fishing communities, over 97% agreed that they eat fish. When asked about the benefits associated with eating fish, 90% claimed to know these benefits. The major benefits of eating fish was that it acted as source of food. When the respondents were asked about the trends of fish consumption the majority agreed that the fish catches were going down hence less and less fish for consumption. The other attributes associated with fish consumption including the species consumed are displayed in Table 1.

**Table 1.** Perceived benefits about fish consumption

Category	Attribute	Frequency	Percentage (%)	Sample *n*
Common benefits of fish consumption	Food	183	85.1	215
	Omega 3	5	2.3	
	Good for heart	6	2.8	
	Satisfaction	6	2.8	
Fish commonly eaten at landing sites	*Lates species*	48	29.8	161
	*Tilapia species*	73	45.3	
	Pelagic fish	25	15.5	
Trend of fish consumption	Increased	5	2.5	198
	Decreased	189	95.5	
	Normal	4	2.0	

#### 3.1.3. Safety, sources of contamination and awareness attributes

When the fishing community household heads was asked about the safety of fish eaten, 92% perceived no known risk associated with consumption and therefore thought it is safe. The major reason behind fish being safe for consumption was that it is always fresh, sweet and has no known drugs. The majority lacked information about contaminants found in fish. The perceived sources of contamination, contaminants available, and safety information is displayed in Table 2.

**Table 2.** Perceived risks associated with fish consumption

Category	Attribute	Frequency	Percentage (%)	Sample *n*
Reasons why fish is safe	Always good/no problems/fresh/sweet/no drug/natural	183	72.9	251
	Eaten them for years	17	6.8	
Awareness about fish contaminants	No	182	76.2	239
	Yes	57	23.8	
Common contaminants aware of	Chemicals	12	4.4	270
	Oil sips	13	4.8	
Sources of the contaminants	People/Fishermen	20	7.3	273
	Oil sips	17	6.2	
	Lake bed	21	7.7	

#### 3.1.4. Relationship between fish consumption and perceived benefits and risks

After establishing the demography and frequencies of the respondents, perceived benefits and risks, there was need to establish the factors which are associated to fish consumption. These factors were generated using *χ* ^2^ analysis and interpretations made at *p* < 0.05. Out of the many factors, ten were statistically associated with weekly fish consumption. and these are reported in Table 3.

**Table 3.** Perceived benefits and risks associated with weekly fish consumption

Attribute	*χ*^2^	Degree	*P* Value
Education	28.9	2	< 0.0001
Awareness about benefits fish consumption	17.3	1	< 0.0001
Methods for preparation	37.1	5	< 0.0001
Parts consumed by children < 5 years	27.4	9	0.001
Parts consumed by children > 5 years	24.6	9	0.003
Parts consumed by Adults	67.6	8	< 0.0001
Preference as reason for fish consumption	25.4	1	< 0.0001
Safety of the fish	32.2	1	< 0.0001
Whether they have heard about fish contaminants	13.7	1	< 0.0001
Fishing as an income generation activity	7.0	1	0.008

The family heads understood that the benefits associated with fish consumption included household sizes, awareness, methods of preparation and lack of knowledge about the fish management organizations.

#### 3.1.5. Modeling of the factors related to household weekly fish consumption

After the bivariate analysis above, the 10 factors were inserted into general linear model of the Poisson family with frequency of consumption as the dependent variable. This generated output of the five factors associated with weekly frequency but in addition it also generated the direction of the research. The factors which increased weekly frequency of fish consumption were lack of awareness about benefits of fish consumption and the number of adults in the family. On the other hand, those factors which decreased weekly fish consumption were method of preparation and lack of awareness about organizations which monitor fishing on the landing sites. The model output was reported in Table 4.

**Table 4.** Model output for the factors related to household weekly fish consumption

Parameter	*β*	Std. Error	95% Confidence interval		*P* value
			Lower	Upper	
(Intercept)	15.232	4.9925	5.447	25.018	0.002
Not aware about benefits	1.571	0.7676	0.066	3.075	0.041
Method of preparation Boiling	−9.548	4.0629	−17.511	1.585	0.019
Method of preparation frying	−10.733	4.1677	−18.901	−2.564	0.010
Adults seven in the family	7.194	3.0820	1.154	13.235	0.020
Not aware about monitoring organizations	−2.961	1.3595	−5.625	−0.290	0.029

**Model information**

**Table:** 

Akaike’s Information Criterion (AIC)	420.036
Bayesian Information Criterion (BIC)	461.751
Consistent AIC (CAIC)	479.751

Large household and lack of awareness about perceived benefits are commonly associated with fish consumption on the landing site and this was witnessed by Barges (2008) when looking at the Canadian situation of fish mercury levels in the local communities. The decrease in consumption was confirmed by the message sent out i.e. should be balanced regarding the benefits and risks associated with fish consumption (Driscoll, Sorensen, & Deerhake, 2012) and contextual (Mansilla-Rivera & Rodríguez-Sierra, 2011). The vulnerable fishing community associated the culinary methods of fish with reduced consumption due to reduced benefits. This was confirmed by Kalogeropoulos et al. (2012) who found out that the method of preparation of fish i.e. frying and grilling instead concentrates the amounts of lead and mercury if present in the fish as compared to boiling. Therefore, the community needs to be sensitized about boiling of fish a method which confers the cardiovascular properties desired from fish as observed by Mozaffarian et al. (2003) in elderly persons in the USA. Verbeke et al. (2008) who looked at fish contribution towards health and the demand for it across Europe especially Belgian consumers showed the need for a balanced message coupled with both benefits and risks of fish consumption. During the survey, only 2.8% of the respondents acknowledge the health benefit, and this shows an information gap.

### 3.2. Qualitative

The decreased fish catches have led the households to believe there is a steady decrease in the fish in the lake. This perspective is also held by key informants, “for some years, the fish has reduced because initially, we used to enjoy fish and really you would buy a lot and really enjoy it but today the fish has reduced in terms of species types and also prices have increased”. Further reduction in fish consumption occurs in fishing communities in a presence of a fish consumption advisory.

However, the household heads and key informants perceived benefits and risks differently in the fishing community. The key informants perceived benefits relate with quality of life. The most commonly perceived benefit of fish consumption is nutrition. The perceived nutrition is associated with food (85.1%) and nutrients i.e. omega three (2.3%). Fish is a staple for fisherfolk. One of the key informants stated that “ ‘Bagungu’ [tribe] almost they depend on fish as food” and without it a whole group of persons will have lost their lifestyle. So these people relate the eating of fish to life style. These nutrition benefits extend to the wellbeing of the community. This food benefit is explained in the statement, “those who take fish, they are looking better meaning that fish has more good values”. This food benefit is in agreement with Burger and Gochfeld (2009) who looked at the American populations who eat fish regardless of whether it was for subsistence or recreation and noted that the perceived benefits from fish consumption range from nutritional to medicinal. These presumed dietary benefits conferred to the fishing community and recreation group according to Burger (2000) is prey to the fish contaminants from community water sources. Clearly, there is need for evidence-based information to relate these perceived benefits to fish consumed in the community.

Growth of infants, “fish for milk the Ngara for especially women who are breast feeding if you want to increase milk when you have delivered, breast feeding, they take that one” and children, “it has some nutrients and that the young children look nice and with ‘muziri’ [sliver fish] the children are able to grow” in the fishing community. Closely tied to the nutrition benefits is the growth which the fishing community was not able to explain. They observed that children who ate fish grew well and appeared healthy. These was confirmed by (Loewe, 2012) in a vulnerable population where growth of children is key for the realization of Sustainable Development Goals of decreased malnutrition and mortalities of children under five. Also, factors that curtail the perceived benefits from fish consumption for the infants and children in fishing communities like drinking water sources and hygiene should be part of fish consumption advisory (Ssebisubi, 2013).

Next perceived benefit that arises out of fish consumption is that of good health, “fish is really good for our health and really many people benefit out of it” and this cuts across all age categories. This perceived health benefit is responsible for the globalization of fish. Closely associated with health is the perceived medicine role. The community associated fish consumption with the treatment i.e. measles, “Mukene the one she was talking about, they were using it for measles” and other medical benefits associated with hastened recovery after delivery in women, “testimony from one lady who has confessed that it helped her recover from those effects, pains in the uterus, back pains, so the ngasa”*.* Unfortunately, it is a fallacy that measles is cured or prevented by consuming fish. Therefore, this misconception needs to be corrected using a fish consumption advisory. As for improved recovery after birth, fish provide a lot of essential nutrients which can aid immune boosting, wound healing and recovery a fact endorsed by Burger and Gochfeld (2009).

Fish is perceived as income and suitable for all situations where money is required for the transaction. The fish resource is in such high demand that the market comes way down to the shore line and even in the waters as stated by the key informant, “there came in a boom of these vehicles moving up to down the wells so they buy the fish in fact when they are still in water”. However, the accrued income gives forth to prostitution, the fish transaction for sex, bars and hotels and then a cycle of poverty typical of the fishing communities (Béné & Merten, 2008; Geheb et al., 2008; Olale & Henson, 2013; Ssebisubi, 2013). Over 95.5% of the respondents acknowledge decreased fish catches in the Lake Albert waters. These dropped catches translate into less income for the fishing community hence the need for revenue diversification. Income-generating skills i.e. trade and other non-fish linked business are key so as to avoid overfishing, “For income, people derive their livelihood on selling fish”(Olale & Henson, 2013). Information on income diversification is vital in a fishing community to sustain the fisheries stock. On the other hand, the increase in fish prices have enabled the fishing community to send the children to school as stated by the key informants, “able to educate the children; some have even been able to buy plots of land” Child education will transform the fishing community through income diversification and development. The education of children in the fishery communities is constrained by few schools on the landing sites, long distances to school and inadequate teaching facilities (Ssebisubi, 2013).

Lastly, fish consumption is perceived to increase potency or manhood, “the potency of men by taking the other species ‘(omutunta)’ [fish]”and libido in men, “fish which I do not remember but will ask the fisheries man, it increases the libido of men”. Fish species “Omutunta” scientifically *Polypterussenegalussenegalus* is rare but delicacy for men. No literature was available to explain this perceived benefit hence there is need for an investigation. The studies by Béné and Merten (2008) and Ssebisubi (2013) explain why HIV is rampant amongst the fishing communities in spite of awareness and control strategies present in the fishery communities.

The personal risks associated with fish consumption included: Stigma and Ill-health. The challenge that stigmatized fish consumption arose from the shape of the fish, number of bones it has, the smell of the fish and resultant effect post consumption of the fish. There is a perception that eel-shaped fish are not suitable for consumption and this include species like the lungfish. The informants who elaborate that, “There is some fish which looks like a snake Laughs Mamba/Lung fish”. Promotion of lungfish consumption in spite of the documented benefits would most likely yield poor responses. Therefore, when designing fish consumption advisories, we should ensure that the species included (even if loaded with mercury and Lead) are in tandem with the fish consumption habits of the community.

One of the informants who observed that, “The children that we have produced do not want to eat fish because they are no longer attractive, they are too small” and this behavior is unbearable for fish consumption is a lifestyle. This practice exhibition is partially confirmed by Johnston and Snow (2007) who looked at people’s life styles in the US especially those with fishing permits in Wyoming and realized that fish consumption for children is promoted because of the benefits offered by fish and in this case omega three. Wheatley and Wheatley (2000) also experienced a similar situation when dealing with fishing communities in Brazil and Canada and called for an approach which linked the risk data to social aspects to gain acceptance. The children in the above countries especially the young ones less than five years were not eager to eat due to the small size of the fish. Sometimes, the children also do want to eat fish because of the high bone to flesh ratio. Failure to eat fish in Brazilian communities where access is not the issue as observed by Mitterer-Daltoé, Latorres, Queiroz, Fiszman, and Varela (2013) among the Brazilian population where awareness campaigns were required to promote fish consumption. The community preferred to export the fish and consume something else.

Fish consumption is associated with ill health i.e. one of the informants reported that, “there are some fish like kasirubana which when eaten you vomit after eating the fish”. Others also added to this situation that, “Somebody eats fish and after a short while gets a running stomach and that is mainly due to poor handling of the fish” The informant made it clear that the group that vomits is the young children. Therefore, other possible causes of ill health like worm infestation and schistosomiasis (elephantiasis) should be investigated. The prevalence of schistosomiasis (elephantiasis) is high around landing sites and causes nausea (Ssetaala et al., 2012).

## 4. Conclusion

The study took place in a vulnerable fishing community of a developing country and looked at the individual perceptions associated with fish consumption. These individual perceptions were expressed as perceived benefits and risks.

The perceived benefits of eating fish in fishing community in third world were: Health, income, manhood and nutrition. These are influenced by household size, awareness about fish management organizations and method of preparation.

The perceived risks associated with fish consumption in fishing community were: Stigma and ill health. These are influenced by the message and hazard levels in fish.

Based on the above, we recommend:

A study to investigate the role of fish consumption in sexual drive for men in fishing community

Creation of an awareness campaign about health benefits and fallacies of fish consumption in fishing communities.

The knowledge on individual perceptions associated with fish consumption will aid fishing community’s members increase fish consumption but with less risks in order to harness the desired benefits.

### 4.1. Limitation and strengths

The key strengths of this work is that it is the first of its kind in the region, and will go a long way in informing research around the fishing villages. Being a mixed study, it also reduces on the shortfalls attributed to either qualitative or quantitative study designs. Due to the fact that this study is executed in a developing country, it will offer insights about the context of vulnerable communities especially fishing communities in aspects of fish consumption.

The limitation could be attributed to the use of a structured questionnaire which was standardized for fishing communities in Uganda and not worldwide.

## Public Interest Statement

Fish consumption is a way of life for fishing communities worldwide. This practice is associated with benefits and risks. Unfortunately, very few people are aware of the advantages and risks related to eating fish. Lack of information about the associated advantages and risks in a fishing community might lower fish consumption amounts. The study, therefore, set out to establish the advantages and risks related to fish consumption in the fishing community. The fishing community listed health, income, nutrition and sex improvement as the benefits of fish consumption. The risks were sickness and dislike of small fish. The advantages and risks depended on the family size, cooking method and awareness about fish monitoring organizations. Therefore, there is the need for a sensitization campaign about the fish hazards, decreased consumption of fish with a high level of pollutants, and to identify the benefits of fish consumption.
